# The Effect of Combined Balance Exercise on Knee Range of Motion, Balance, Gait, and Functional Outcomes in Acute Phase Following Total Knee Arthroplasty: A Single-Blind Randomized Controlled Trial

**DOI:** 10.3390/medicina60091389

**Published:** 2024-08-24

**Authors:** Jungae An, Seong-Jin Cheon, Byoung-Hee Lee

**Affiliations:** 1 Graduate School of Physical Therapy, Sahmyook University, Seoul 01795, Republic of Korea; ajapt@syu.ac.kr (J.A.); gis9000@naver.com (S.-J.C.); 2 Department of Physical Therapy, Sahmyook University, Seoul 01795, Republic of Korea

**Keywords:** range of motion, function, balance, gait, total knee arthroplasty

## Abstract

*Background and Objectives*: Total knee arthroplasty (TKA) improves balance performance in patients with osteoarthritis; however, balance deficit and fall incidence after TKA have been reported. This study aimed to determine the effects of combined balance exercises on knee range of motion (ROM), balance, gait, and functional outcomes during the acute phase after TKA. *Materials and Methods*: A total of 42 participants were randomly assigned to either the combined balance group (*n* = 21) or the general physical therapy (control) group (*n* = 21). The combined balance exercise group performed exercise programs for 30 min per session, five times a week for 4 weeks (20 sessions), and the control group completed general physical therapy, which included active simple exercise. Measurements were performed before and after the 4 weeks of training to assess changes in the Western Ontario and McMaster Universities Osteoarthritis Index (WOMAC), knee joint ROM, static balance, timed up-and-go (TUG), and 10 m walk test (10 MWT). *Results*: The combined balance exercise group demonstrated significant improvements post intervention (*p* < 0.05) for all outcomes. The time × group interaction effect for the WOMAC scores showed statistically significant interaction effects for pain, stiffness, and physical function; the static and dynamic balance values showed statistically significant interaction effects for CEA, PL, AV, and TUG; and gait ability showed a significant interaction effect for the 10 m walk test (*p* < 0.05). *Conclusions*: This study confirmed that combined balance training with general physical therapy has a positive effect on ROM, static and dynamic balance, gait, and functional outcomes in the acute phase post TKA and that combined balance exercise can be proposed as a rapid rehabilitation intervention with general physical therapy following TKA.

## 1. Introduction

Osteoarthritis, a chronic degenerative disease associated with aging, causes the deterioration of joint cartilage, leading to pain, stiffness, and limited functional movement of the joints [1]. General risk factors for the development of osteoarthritis, including aging, obesity, and female sex, are associated with underlying causes and structural changes [2]. Additionally, metabolic and mechanical factors such as smoking, genetics, and joint injuries contribute to its occurrence; these factors commonly contribute to osteoarthritis, particularly affecting weight-bearing joints such as the knee, and they influence structural aspects including muscle weakness, joint instability, joint deformity, pain, and stiffness [3,4,5].

Total knee arthroplasty (TKA) is performed to alleviate pain and improve physical function when severe pain and persistent knee joint deformities impair physical function and affect daily life [6,7]. However, after successful surgical procedures, 37% of TKA patients experience limitations in their physical function in the first year post TKA and only 36% of TKA patients engage in physical and sports activities preoperatively [8,9]. During the TKA procedure, the anterior cruciate ligament, joint cartilage, and meniscus are removed and replaced by the prosthesis. The removed structures are sensory receptors that influence joint stability, proprioception, motor control, and balance [10,11]. Moreover, after TKA, genu valgus or genu varus deformities caused by osteoarthritis are realigned, creating new tension in the lower extremities [12]. Following TKA, walking ability may decrease due to the reduced ability to move the body forward, restrictions in knee flexion and ankle dorsiflexion, shorter step length, prolonged double-support time, and shortened single-support time, leading to changes in the gait pattern during walking [13,14]. Additionally, TKA patients exhibit a 16–22% decrease in walking speed compared to non-surgical patients, and most TKA patients show a decreased range of motion during the gait cycle [15,16]. One month after surgery, there is a reduction in the contraction and cross-sectional area of the quadriceps femoris muscle, with a decrease in strength of up to 60%, and the strength of the knee extensor muscles plays a crucial role in determining both static and dynamic balance abilities by aiding in proprioception and joint range of motion, thereby reducing postural sway [17,18,19].

Static and dynamic balance refers to the body’s response to sensory input and its ability to maintain equilibrium in a stable posture while performing tasks or during locomotion [20,21]. Balance is the most fundamental and essential factor for maintaining posture and performing activities such as walking in one’s daily life [22]. However, due to aging and knee osteoarthritis, balance and mobility abilities decline significantly, leading to a higher incidence of falls [7,23]. Falls can result in various complications in elderly individuals, including fractures, traumatic brain injuries, and other comorbidities. In severe cases, falls can be fatal accidents [24]. Balance training in seniors at risk of falls has been shown to prevent falls and significantly improve their walking and balance abilities [19]. Although TKA improves balance performance in osteoarthritis, fall incidence after TKA has been reported [25,26].

Balance training has been applied singly or in combination with other interventions for the rehabilitation of patients undergoing TKA. Balance training promotes the recovery of proprioception and joint instability from damaged joint capsules due to TKA [27]. Previous studies have reported that balance training increases the range of motion (ROM) of the knee and muscle strength and improves walking ability and physical function [28,29,30,31]. However, static and dynamic components are not adequately addressed, and the types, methods, and intervention duration of balance training vary [32]. Conventional early rehabilitation for TKA focuses on pain control, optimization of ROM, and bed mobility; however, the effect of a rapid rehabilitation program with balance training for the acute phase post-TKA on functional recovery, balance, and gait has not been reported [33,34]. Therefore, this study aimed to investigate the effects of combined balance exercises on ROM, balance, gait, and functional outcomes in patients in the acute phase following TKA.

## 2. Materials and Methods

### 2.1. Participants

The participants were 42 patients who underwent unilateral TKA and were admitted to an orthopedic surgery and rehabilitation hospital. Before recruiting participants for this study, we performed a power analysis using G*Power version 3.1.9.7 (Franz Faul, University Kiel, Kiel, Germany, 2020). Using repeated-measure ANOVA and studying the within–between interactions, an effect size f of 0.25 was obtained for all the outcome measures, with an α error probability of 0.05, a power 1-β error probability of 0.95, two groups, and four measurements. Because the estimated target sample size was 36, we considered a dropout rate of 10–15% and recruited 42 participants [31]. 

The inclusion criteria were as follows: patients who underwent unilateral cemented TKA; could walk independently at least 10m. The exclusion criteria were as follows: patients who underwent revision TKA; those who had a history of lower-extremity surgery within the past 6 months; patients aged 80 years and older; patients with neurological conditions (stroke, dementia, and Parkinson’s disease); those with conditions such as rheumatoid arthritis affecting the knee joint’s range of motion or physical function; individuals with psychiatric disorders; those without a history of spine-related surgery; and individuals who declined consent.

### 2.2. Ethical Statement

All the participants signed a consent form after the procedure, and the purpose of this study was explained to them. This study was approved by the Sahmyook University Institutional Review Board (approval number: 2-1040781-A-N-012021054HR; approval date: 28 June 2021) and the Clinical Research Information Service (KCT0006762). The participants fully understood the objectives and procedures used in this study. This study adhered to the ethical principles of the Declaration of Helsinki.

### 2.3. Study Design

A single-blinded, randomized, controlled trial design was used for this study.

This study obtained mandatory medical record review approval before the experiment. Clinical characteristics such as medical history, present illness, surgical date, symptoms, and general characteristics, including age, height, and weight, were collected from all the study participants. Initially, 42 participants underwent pre-screening. To minimize the errors associated with the experiment, the participants were randomly assigned to two groups using the Research Randomizer program (http://www.randomizer.org/ (accessed on 17 July 2021)). The groups were divided into a CBE (combined balance exercise) group (*n* = 21) and a control (general physical therapy exercise) group (*n* = 21). The patients in each group were unaware of their group assignments. The intervention and assessment were conducted in an independent room, and the participants participated in training according to their schedules. The assessors did not administer the intervention to the experimental group and were completely blinded to which group the patients belonged to. The patients were assessed the day before TKA surgery and after the 4-week intervention. A total of 38 participants were included in the final analysis, and those with a less than 90% participation rate were excluded (three patients withdrew from the study and one patient was discharged).

### 2.4. Experimental Procedure

This study was conducted in an inpatient acute care unit. All TKA procedures were performed using a tricompartmental, minimally invasive, mid-vastus approach with a high-flexion mobile prosthesis (Implantcast; GMBH Lüneburger Schanze, Buxtehude, Germany). The postoperative rehabilitation program was started immediately after TKA, and an early rehabilitation program, including continuous passive motion, intermittent mechanical compression, cold pack, and isometric and active range of motion exercises by the patient, was applied. On the 2nd postoperative day after drain removal, the patients wore compression stockings. Passive and active ROM exercises were started at the bedside, and weight-bearing with a walker was also started. On the 3rd postoperative day, all patients started stretching exercises of the hamstring muscles and strengthening of the hip abductor muscles. 

The CBE group engaged in a combined balance exercise with conventional physical therapy for 30 min per session, 5 times a week, for 4 weeks from the 3rd day after TKA. The control group underwent a hospital protocol for general physical therapy. Additionally, all the participants performed continuous passive motion 20 min after the intervention. The combined balance exercise and physical therapy interventions were conducted by physical therapists with over five years of experience in the field of orthopedic physical therapy. They were knowledgeable about the characteristics of patients who underwent TKA and the potential issues that may arise during the research process. One week before starting the experiment, the physical therapists acquired proficiency during training. The assessors were trained in the use of measurement equipment, and the same assessor performed measurements before and after the intervention.

### 2.5. Training Program

#### 2.5.1. Combined Balance Exercise 

The combined balance exercises were developed based on previous studies [17,35,36,37] and consisted of weekly static and dynamic balance exercises. The combined balance exercises followed the manual provided by the hospital and were conducted under the supervision of three physical therapists. The total exercise time was 30 min, and comfortable walking was performed for 5 min for warm-up and cool-down. The rest time was set to 30–40 s, and when the patient requested, additional time was allowed. Knee braces and walkers were used during the intervention to ensure the safety of the participants (Table 1).

#### 2.5.2. General Physical Therapy 

The general physiotherapy intervention followed the hospital’s manual, initiating simple active exercises three days post surgery. To ensure the participants’ safety, the interventions were performed using knee braces, followed by a walker. The set and rest intervals between the sets were consistent with those in the previous weeks (Table 2). 

### 2.6. Outcome Measures

The knee joint range of motion and Western Ontario and McMaster Universities Osteoarthritis Index (WOMAC) were used to evaluate physical function. The WOMAC is a standardized questionnaire used by healthcare professionals to assess the status of patients with knee and hip osteoarthritis, encompassing joint pain, stiffness, and physical function; WOMAC assesses pain scores ranging from 0 to 20, stiffness scores from 0 to 8, and physical function scores from 0 to 68. Higher scores indicate more severe pain, stiffness, and functional limitations (ICC = 0.92) [38]. The knee joint motion measurements were taken using a digital goniometer (Biometrics, USA, 2008) (ICC = 0.994) [39]. The participants performed maximum flexion while seated for the assessment. The axis of the goniometer was fixed at the lateral aspect of the femur and knee joints, whereas the stationary arm was aligned along the lateral aspect of the thighbone. The moving arm was positioned along the midline from the head of the fibula to the ankle to measure the range of motion. To minimize the measurement errors, three repeated measurements were performed. The average of these measurements was used for the analysis. 

Static balance was assessed using a force plate (FDM multifunction force measuring plate; Zebris Medical, Isny, Germany, 2016). The force plate utilized individual pressure sensors (force sensors) arranged at a density of one sensor per 1 cm^2^ to analyze the distribution of static and dynamic forces on the lower limbs while standing still or walking. The reliability of the force plate measurements ranged from ICC = 0.83 to 0.99 [40]. For the measurement procedure, the participants stood on the force plate facing forward. The measurements, including the confidence ellipse area (CEA), swaying distance, and swaying velocity, were repeated three times, and the average values were used for the analysis. Dynamic balance was assessed using The Timed Up and Go (TUG) test. TUG is utilized to evaluate functional mobility and walking ability in elderly individuals with conditions such as stroke, Parkinson’s disease, or arthritis [41]. The reliability of the TUG test showed an intraclass correlation coefficient (ICC) of 0.91–0.92 [42]. The measurement procedure involved the participants starting from a seated position on a chair at a height of 50 cm. Upon the examiner’s signal, the participant stood up, walked forward for 3 m, turned around, and returned to sit on the chair [43]. The time taken to complete this task was measured, and the process was repeated three times. The average values were then calculated.

Gait was assessed using a 10 m walking test. The 10 m walking test involved measuring the time taken for the participant to walk a distance of 10 m on a flat surface. This test was repeated three times, and the average value was calculated. The test demonstrated reliability with an intraclass correlation coefficient (ICC) of 0.92–0.97 [44].

### 2.7. Data Analysis

All statistical analyses were performed using SPSS ver. 28.0 (SPSS Inc., Chicago, IL, USA, 2021) to calculate the means and standard deviations. The normal distribution of the variables was confirmed using the Shapiro–Wilk test for all the participants. Descriptive statistics were used to describe the general characteristics of the participants, and an independent *t*-test was conducted to examine homogeneity between the groups. Paired sample *t*-tests were performed to compare the pre- and post-intervention differences in the dependent variables within each group. In contrast, independent sample *t*-tests were used to investigate the differences between groups. A two-way analysis of variance (ANOVA) was used to assess the interaction between group and time. The effect sizes were presented as partial eta-squared (η^2^p). We described the threshold values for the effect sizes as 0.01 (small), 0.06 (medium), and 0.14 (large), following Cohen [45]. The statistical significance level for all the data was set at *p* < 0.05. 

## 3. Results

**Figure 1:**
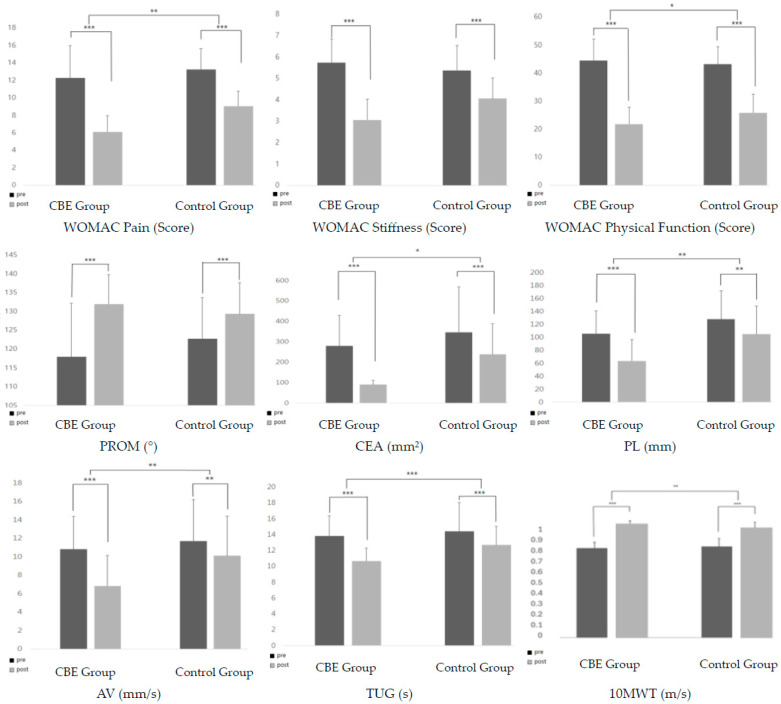
Study variables: within–between group changes. CBE = combination balance exercise group; WOMAC = the Western Ontario and McMaster Universities Osteoarthritis Index; PROM = passive range of motion; CEA = confidence ellipse area; AV = average velocity; PL = path length; TUG= Timed Up and Go test; and 10 MWT= 10 m walk test. * *p* < 0.05; ** *p* < 0.01; and *** *p* < 0.001.

A total of 38 female patients with unilateral TKA completed this study. The participants’ general characteristics and homogeneity test results are presented in Table 3. No significant differences were observed between the groups. Figure 1 presents the within-group changes in all the outcomes.

Physical function was evaluated using the WOMAC scores and the knee joint range of motion. The pre-and post-exercise changes in physical function after four weeks of exercise in the experimental and control groups are shown in Table 4. The time × group interaction effect for the WOMAC scores was statistically significant, with the following *F* values—*F*(1.36) = 6.523, *p* = 0.015 for pain; *F*(1.36) = 11.814, *p* = 0.001 for stiffness; and *F*(1.36) = 4.879, *p* = 0.034 for physical function—and a significant effect of time for all the WOMAC scores (*p* < 0.001). However, in the group effect examination, only the pain score showed a significant difference, with *F* values of *F*(1.36) = 17.307 and *p* = 0.010. 

In the range of motion of the knee, a significant improvement was observed in the CBE group, from 117.95° before exercise to 131.95° after exercise (*p* < 0.001). In the control group, a significant improvement was observed from 122.68° before exercise to 129.32° after exercise (*p* < 0.001). In the comparison of time points before and after exercise, significant differences were found in both the time effect, with *F* values of *F*(1.36) = 47.505, *p* < 0.001), and the time × group interaction, with *F* values of *F*(1.36) = 6.059, *p* = 0.019).

Static balance ability was measured using a force plate. Table 5 presents the results. The time × group interaction effect for the static balance values showed statistically significant interaction effects, with the following *F* values—*F*(1.36) = 4.175, *p* = 0.048 for CEA; *F*(1.36) = 17.464, *p* = 0.010 for PL; and *F*(1.36) = 7.564, *p* = 0.009 for AV—and a significant effect of time on all the static balance values (*p* < 0.001). In the group effect, CEA with *F* values of *F*(1.36) = 5.407, *p* = 0.026, and PL with *F* values of *F*(1.36) = 17.464, *p* = 0.010, showed a significant difference. The dynamic balance was assessed with the TUG test. The CBE group showed a significant improvement, from 13.83 s before exercise to 10.67 s after exercise (*p* < 0.001). In the control group, a significant improvement was observed from 14.39 s before exercise to 12.67 s after exercise (*p* < 0.05). A statistically significant time × group interaction effect was observed for the TUG test time, with *F* values of *F*(1.36) = 7.347, *p* = 0.01.

Gait ability was assessed using the 10 m walk test. The results are summarized in Table 6. A significant improvement was observed in the CBE group from 12.3 s before exercise to 9.92 after (*p* < 0.001). In the control group, a significant improvement was observed from 12.06 s before exercise to 11.40 s after (*p* < 0.05). A comparison of the interaction effects showed a statistically significant time × group interaction effect for the 10 m walk test time, with *F* values of *F*(1.36) = 4.288, *p* = 0.046, and a significant time effect (*p* < 0.001). However, no significant differences in gait ability were observed between groups.

## 4. Discussion

Early rehabilitation after TKA typically involves ROM and muscle strength exercises because of their safety. To enhance the functional abilities necessary for daily life, it is important to focus on balance, strength, and proprioception, and various rehabilitation exercises are required to restore functional exercise performance [46]. This study found that 4-week combined balance exercises improved the knee ROM, balance, gait, and physical outcomes of acute-phase TKA. The effects of early postoperative rehabilitation programs have also been reported [47]. However, the incorporation of combined balance exercises in early rehabilitation programs has rarely been reported. In this study, the patients started combined balance exercises three days after TKA.

Similarly, Jogi et al. conducted a typical exercise with balance training for 5 weeks; the participants started these exercises 7–10 days after TKA and reported an improvement in balance and functional mobility [31]. In this study, we compared the changes after the WOMAC scores showed significant improvements in pain, stiffness, and physical function. The WOMAC index has been reported to be an accurate measurement method for assessing TKA outcomes. The minimum clinically important difference (MCID) for WOMAC after TKA was suggested to be 11 points for pain, 9 points for function, 8 points for stiffness, and 10 for the total score [48]. The difference in WOMAC in the CBE group was 6.16 points for pain, 22.68 points for physical function, 2.68 points for stiffness, and 33 points for the total score. Although MCID was not achieved in some subscales of the WOMAC, the presented MCID corresponds to one year after TKA surgery and, to our knowledge, has not been reported for patients with early-phase TKA. In a study by Jogi et al., WOMAC physical function improved by 23 points, which is similar to our results. The range of motion in the CBE group significantly increased from 117 to 131 pre–post intervention compared to the general physical therapy group. This combined balance exercise included standing on one leg while holding onto a walker, performing up-and-down movements while holding onto a walker, and walking with high knee lifts. These exercises are designed to enhance proprioception; however, they are also believed to significantly improve muscle strength, which may contribute to the observed significant effects on the joint range of motion [49].

Piva et al. [17] applied functional training combined with balance exercises to patients undergoing TKA. They noted clinically significant improvements in lower-limb balance and gait abilities. In this study, the CBE group showed significant improvement in their dynamic and static balance abilities, which was achieved by implementing a combined balance exercise program which included movements to strengthen the knee extensor muscles and repetitive training such as walking with high knee lifts, standing on one leg while holding onto a walker, walking in a figure-eight pattern, and performing up-and-down movements while holding onto a walker. This suggests that such training activates proprioception, sensory perception, and motor responses while enhancing knee stability and muscle coordination in patients [50]. Furthermore, the results of this study showed that the time × group interaction effect on the static postural balance values was statistically significant for CEA, AV, and PL. The dynamic balance was measured using TUG. The TUG time in the CBE group decreased from 13.83 s to 10.67 s after intervention. The TUG test is commonly used as a screening tool to predict fall risk, and the average time for elderly women aged 70–79 years is reported to be 9 s (5–13 s) [51]. Combined balance exercises improve the muscles of the lower extremities and enhance proprioception and the activation of the core muscles. This improvement in coordination and muscle activation likely reduces the sway range during quiet standing and walking, thereby providing positive effects. Therefore, continuous combined balance exercises are necessary for patients undergoing TKA to achieve an independent return to daily activities and the recovery of balance function.

After TKA, patients often exhibit a slow walking speed, a decreased step length, a reduced knee flexion angle during the swing and stance phases, and sustained simultaneous contraction of the quadriceps, hamstrings, gastrocnemius, and tibialis anterior muscles, leading to altered gait patterns [52,53]. In daily life, walking abilities are needed in various environments, including walking on flat surfaces, crossing thresholds, and navigating stairs both indoors and outdoors; these movements involve navigating or avoiding obstacles continually. While individuals with normal gait patterns can easily perform these continuous movements, patients with abnormal gait patterns may find it difficult to execute them, increasing the likelihood of frequent falls [54,55]. Patients undergoing TKA experience reduced lower-limb muscle function, resulting in a decreased walking speed and an increased susceptibility to environmental influences. Therefore, balance and proprioception-enhancing exercises should be implemented to improve environmental factors and walking ability [56]. Studenski et al. [57] stated that, to achieve normal walking, a speed of 1.0 m/s to 1.3 m/s is necessary. As in previous research, we measured the time taken during performance using the 10 m walk test to examine changes in the gait variables. In the 10 m walk test, the CBE group increased from 0.813 m/s to 1.00 m/s, while the general physical therapy exercise group also significantly decreased from 0.79 m/s to 0.88 m/s. In the gait cycle, the steps related to walking speed are those which take place from the mid-stance phase to the late and late-stance phases. During these gait phases, the main activated muscles include the gluteus maximus, the quadriceps, and the calves [58]. The combined balance exercises in our study included standing on one leg while holding onto a walker, standing on one leg while performing up-and-down movements, walking while lifting the knees high, stepping up and down on a platform, and other exercises in our study. These exercises promoted the activation of the gluteus maximus, the quadriceps, and the lateral hip rotator muscles on the affected side. By incorporating exercises such as heel raises and walking while lifting the heels, not only the function of the knee joint but also the activation of the gluteus medius and hamstring muscles of the hip joint during walking can be enhanced, leading to improvements in balance and walking speed.

The limitations of this study include the difficulty in interpreting the findings, as the effects of the intervention were observed in a group consisting solely of female patients aged 60–70 who had undergone TKA. The study sample was small and recruited from only one medical institution in a specific region. Additionally, the short duration of the 4-week exercise intervention only allowed for short-term effects to be assessed, making it challenging to demonstrate the long-term effects of interventions lasting > 4 weeks. Furthermore, the combined balance exercise was performed for 4 weeks starting 3 days after TKA, and there was a lack of control over individual medication dosages for patients in the acute postoperative period, as they were not consistently regulated according to the hospital protocols.

Future research should focus on conducting long-term multicenter studies and follow-up observations of patients who have undergone TKA with a large sample size to determine the long-term effects of exercise interventions. Additionally, repeated studies are needed to expand the target population of physical therapy by incorporating combined balance exercises and establishing evidence supporting their necessity.

## 5. Conclusions

This study confirmed that general physical therapy incorporating combined balance exercises has beneficial effects on knee ROM, static and dynamic balance ability, gait, and physical function in patients undergoing acute-phase TKA. This finding provides evidence for improving functional ability in daily life, preventing falls, and enabling functional walking. Therefore, general physical therapy that incorporates combined balance exercises is an effective intervention for early TKA rehabilitation.

## Figures and Tables

**Table 1:** Combined balance exercise.

Week	Exercise Program			
1st week	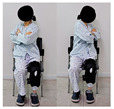	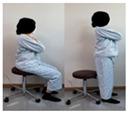	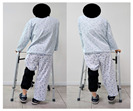	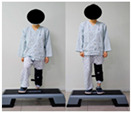
Exercise	Seated torso rotations	Sit to stand	Shift weight to one side	Step-ups and step-downs
Practice	Each 10 reps/2 sets	Each 10 reps/2 sets	Each 10 reps/2 sets	Each 10 reps/2 sets
2–3rd week	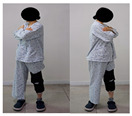	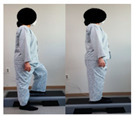	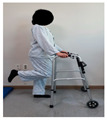	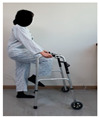
Exercise	Trunk rotations while standing	Side step-ups and downs	Standing on one leg while holding onto a walker	Lifting knees high and walking forward
Practice	Each 10 reps/2 sets	Each 10 reps/2 sets	Each 10 reps/2 sets	Each 10 reps/2 sets
4th week	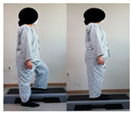	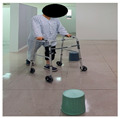	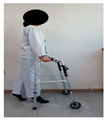	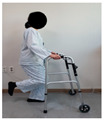
Exercise	Side step-ups and step-downs	Figure-eight walking	Heel raises while walking with a walker	Using a walker to perform up-and-down movements
Practice	Each 10 reps/2 sets	Each 1 min/2 reps	Each 1 min/2 reps	Each 10 reps/2 sets

The total exercise time is 30 min, and the rest time between sets is 30–40 s.

**Table 2:** General physical therapy.

Week	Exercises	Practice
1–2nd week	Knee range of motion exercises in a seated position Leg extension exercises while lying down Leg lifting exercises while lying down Ankle dorsiflexion/plantarflexion exercises	Each 5 s/10 reps/5 sets
3–4th week	Knee range of motion exercises in a seated position Knee extension exercises with resistance in a seated position Knee flexion exercises while standing Knee extension with resistance while standing Hip abduction exercises while standing.	Each 5 s/10 reps/5 sets

The total exercise time is 30 min, and the rest time between sets is 30–40 s.

**Table 3:** General and medical characteristics of the participants (*N* = 38).

Characteristics	CBE Group (*n* = 19)	Control Group (*n* = 19)	*X*^2^/*t* (*p*)
Age (years)	73.6 (4.8) ^a^	72.3 (4.6)	0.865 (0.393)
Affected side (Rt/Lt)	8/11	10/9	0.422 (0.516)
Height (cm)	154.8 (3.9)	152.5 (7.1)	1.248 (0.222)
Weight (kg)	63.7 (7.9)	60.5 (8.6)	1.185 (0.244)
BMI (kg/m^2^)	26.5 (2.6)	25.9 (3.0)	0.585 (0.562)
BMD (T-score)	−2.3 (1.2)	−3.3 (2.4)	1.562 (0.127)
Hypertension, N (%) (Yes/No)	14 (73.7%)/5 (26.3%)	12 (63.2%)/7 (36.8%)	0.487 (0.485)
Diabetes mellitus, N (%) (Yes/No)	11 (57.9%)/8 (42.1%)	10 (52.6%)/9 (47.4%)	0.106 (0.744)
Hyperlipidemia, N (%) (Yes/No)	13 (68.4%)/6 (31.6%)	9 (47.4%)/10 (52.63%)	1.727 (0.189)
Depression, N (%) (Yes/No)	3 (15.8%)/16 (84.2%)	2 (10.5%)/17 (89.5%)	0.230 (0.631)

^a^ M (SD); CBE = combination balance exercise group; BMI = body mass index; and BMD = bone mineral density.

**Table 4:** Changes in physical function (*N* = 38).

Variable		CBE Group (*n* = 19)	Control Group (*n* = 19)	*p*-Value	Time	Group	Time × Group	
					*F*(*p*)	*F*(*p*)	*F*(*p*)	*η* ^2^ *p*
WOMAC Pain	Pre-test	12.26 (3.72) ^a^	13.22 (2.42)	0.309	197.336 (0.000)	7.307 (0.010)	6.523 (0.015)	0.153
	Post-test	6.11 (1.85)	9.05 (1.68)					
	Mean difference	6.16 (2.77)	4.26 (1.66)	0.015				
	*p*-value	0.000	0.000					
WOMAC Stiffness	Pre-test	5.74 (1.09)	5.37 (1.16)	0.322	100.940 (0.000)	1.290 (0.264)	11.814 (0.001)	0.247
	Post-test	3.05 (0.97)	4.05 (0.97)					
	Mean difference	2.68 (1.29)	1.32 (1.15)	0.001				
	*p*-value	0.000	0.000					
WOMAC Physical Function	Pre-test	44.47 (7.567)	43.11 (6.315)	0.549	264.960 (0.000)	0.578 (0.452)	4.879 (0.034)	0.119
	Post-test	21.79 (5.931)	25.84 (6.585)					
	Mean difference	22.68 (7.18)	17.26 (7.92)	0.034				
	*p*-value	0.000	0.000					
WOMAC Total (Score)	Pre-test	63.95 (7.656)	61.63 (8.908)	0.396	365.912 (0.000)	1.784 (0.190)	12.558 (0.001)	0.259
	Post-test	30.95 (6.811)	38.95 (8.256)					
	Mean difference	33.00 (8.266)	22.68 (9.627)	0.001				
	*p*-value	0.000	0.000					
PROM (°) Knee Flexion	Pre-test	117.95 (14.24)	122.68 (11.01)	0.259	47.505 (0.000)	0.113 (0.738)	6.057 (0.019)	0.144
	Post-test	131.95 (7.89)	129.32 (8.37)					
	Mean difference	14.00 (11.79)	6.63 (5.58)	0.021				
	*p*-value	0.000	0.000					

^a^ M (SD); CBE = combination balance exercise group; WOMAC = the Western Ontario and McMaster Universities Osteoarthritis Index; and PROM = passive range of motion. Level of significance: *p* < 0.05. *F*: two-way repeated-measure analysis of variance.

**Table 5:** Changes in static and dynamic balance (*N* = 38).

Variable		CBE Group (*n*= 19)	Control Group (*n*= 19)	*p*-Value	Time	Group	Time × Group	
					*F*(*p*)	*F*(*p*)	*F*(*p*)	*η* ^2^ *p*
CEA (mm^2^)	Pre-test	279.78 (150.75) ^a^	345.97 (224.43)	0.294	54.618 (0.000)	5.407 (0.026)	4.175 (0.048)	0.104
	Post-test	90.30 (21.24)	238.57 (150.32)					
	Mean difference	189.48 (140.48)	107.39 (104.52)	0.048				
	*p*-value	0.000	0.000					
PL (mm)	Pre-test	105.81 (34.75)	127.78 (44.63)	0.099	86.668 (0.000)	6.644 (0.014)	7.464 (0.010)	0.172
	Post-test	63.86 (32.41)	104.87 (43.36)					
	Mean difference	41.95 (23.17)	22.91 (19.63)	0.010				
	*p*-value	0.000	0.000					
AV (mm/s)	Pre-test	10.80 (3.57)	11.71 (4.51)	0.494	42.337 (0.000)	2.979 (0.093)	7.564 (0.009)	0.174
	Post-test	6.82 (3.34)	10.10 (4.31)					
	Mean difference	3.97 (3.01)	1.61 (2.22)	0.009				
	*p*-value	0.000	0.005					
TUG (s)	Pre-test	13.83 (2.54)	14.39 (3.63)	−0.584	84.502 (0.000)	2.465 (0.125)	7.347 (0.010)	0.169
	Post-test	10.67 (1.63)	12.67 (2.35)					
	Mean difference	3.15 (1.37)	1.71 (1.85)	0.010				
	*p*-value	0.000	0.001					

^a^ M(SD); CBE = combination balance exercise group; CEA = confidence ellipse area; AV = average velocity; PL = path length; and TUG = Timed Up and Go test. Level of significance: *p* < 0.05. *F*: two-way repeated-measure analysis of variance.

**Table 6:** Changes in gait ability (*N* = 38).

Variable		CBE Group (*n*= 19)	Control Group (*n*= 19)	*p*-Value	Time	Group	Time × Group	
					*F*(*p*)	*F*(*p*)	*F*(*p*)	*η* ^2^ *p*
10MWT (m/s)	Pre-test	0.813 (0.69) ^a^	0.79 (0.60)	0.726	40.108 (0.000)	1.769 (0.192)	4.288 (0.046)	0.106
	Post-test	1.00 (0.89)	0.88 (0.74)					
	Mean difference	0.187 (0.60)	0.09 (0.47)	0.046				
	*p*-value	0.000	0.012					

^a^ M(SD); CBE = combination balance exercise group; 10MWT = 10 m walk test. Level of significance: *p* < 0.05. *F*: two-way repeated-measure analysis of variance.

## Data Availability

Data are contained within the article.
